# MdSnRK1.1 interacts with MdJAZ18 to regulate sucrose-induced anthocyanin and proanthocyanidin accumulation in apple

**DOI:** 10.1093/jxb/erx150

**Published:** 2017-05-25

**Authors:** Xiao-Juan Liu, Xiu-Hong An, Xin Liu, Da-Gang Hu, Xiao-Fei Wang, Chun-Xiang You, Yu-Jin Hao

**Affiliations:** 1 State Key Laboratory of Crop Biology, National Research Center for Apple Engineering and Technology, College of Horticulture Science and Engineering, Shandong Agricultural University, Tai-An, Shandong, China; 2 Research Institute of Pomology, CAAS, Xingcheng, China

**Keywords:** Anthocyanins, JA, JAZ protein, MBW complex, proanthocyanins, SnRK1 protein kinase, sucrose

## Abstract

Sugars induce anthocyanin biosynthesis in plants. As a conserved energy sensor, SnRK1 (SNF1-related kinase 1) is involved in sucrose-induced anthocyanin accumulation. However, the exact molecular mechanism by which SnRK1 regulates the biosynthesis of anthocyanins and proanthocyanidins (PAs) in response to sucrose in plants is not clear. In this study, it was found that MdSnRK1.1 interacted with MdJAZ18 protein which acts as a repressor in the jasmonate (JA) signaling pathway. MdSnRK1.1 then phosphorylated MdJAZ18 to facilitate its 26S proteasome-mediated degradation, which released MdbHLH3 thereby activating the expression of the regulatory and structural genes, thus finally promoting the biosynthesis of anthocyanins and PAs. Taken together, these results demonstrate the involvement of MdSnRK1.1 in sucrose-induced accumulation of anthocyanins and PAs. For the first time, our findings shed light on the molecular mechanism by which the crosstalk of sucrose and JA signaling regulates flavonoid biosynthesis.

## Teaser

MdSnRK1.1 regulates anthocyanin and proanthocyanidin accumulation in apple by phosphorylation and modulation of MdJAZ18 stability, explaining how jasmonic acid acts together with sucrose to regulate anthocyanin biosynthesis.

## Introduction

Flavonoids are a class of important secondary metabolites in plants. They mainly include flavonols, anthocyanins, flavones, and proanthocyanins (PAs). Anthocyanins contribute to the colors of flowers and fruit, ranging from blue to red with the purpose of attracting pollinators and seed distributors (Koes *et al.*, 2006). The presence of anthocyanins in fruit mainly determines fruit exterior quality, which is an important consideration in consumer choice (Allan *et al.*, 2008; Li *et al.*, 2012). PAs are present in various organs, particularly in leaf, bark, root, fruit, and seed (Barbehenn and Constabel, 2011). These flavonoids generally exist in the form of colorless polymers, and are oxidized into brown complexes under various stress stimuli (Pourcel *et al.*, 2005). PAs are considered as one of the most effective natural antioxidants responsible for the removal of free radicals from the human body. In addition, anthocyanins and PAs play important roles in resistance to insect attacks and pathogen infection in plants, and are beneficial for human health (Dixon *et al.*, 2005; Scalbert *et al.*, 2005).

Anthocyanins and PAs, also known as condensed tannins, are derived from phenylpropanoids and malonyl-CoA in the flavonoid biosynthetic pathway which contains a series of enzymes (Yoshida *et al.*, 2015). These enzymes are encoded by biosynthetic structural genes which include early biosynthetic genes (EBGs) such as *phenylalanine ammonia-lyase* (*PAL*), *chalcone synthase* (*CHS*), *chalcone isomerase* (*CHI*), *flavanone 3-hydroxylase* (*F3H*), and *flavonoid 3'-hydroxylase* (*F3'H*), as well as late biosynthetic genes (LBGs), including *anthocyanidin synthase* (*ANS*), *dihydroflavonol reductase* (*DFR*), *leucoanthocyanidin oxidase* (*LDOX*), *UDP-Glc:flavonoid 3-O-glucosyltransferase* (*UF3GT*), and *anthocyanidin reductase* (*ANR*) (Solfanelli *et al.*, 2006; Shan *et al.*, 2009; Jeong *et al.*, 2010). The branch of the anthocyanin and PA synthetic pathway exists in the late stage of the flavonoid biosynthetic pathway, mainly involving *ANS*, *DFR*, *LDOX*, and *UF3GT* genes whose products are responsible for the synthesis of anthocyanins, and *ANR* and *leucoanthocyanidin reductase* (*LAR*) whose products are responsible for PA synthesis (Koes *et al.*, 2006). The structural genes are regulated by the WD-repeat/bHLH/MYB complex (MBW), which consists of WD-repeat protein, basic helix–loop–helix (bHLH), and MYB transcription factors (TFs). The conserved MBW regulatory mechanism works in various plant species (Espley *et al.*, 2006; Qi *et al.*, 2011; Albert *et al.*, 2014; An *et al.*, 2015; Yoshida *et al.*, 2015). In apple, an increasing number of MBW members has been proven to be involved in the control of anthocyanin and PA biosynthesis. The bHLH TFs such as MdbHLH3 and MdbHLH33 recruit WD-repeat protein MdTTG1 and the R2R3-MYB TF, MdMYB1, to mediate anthocyanin biosynthesis (An *et al.*, 2012). MdbHLH3 interacts with MdMYB9 and MdMYB11 to promote anthocyanin and PA accumulation by binding to the promoter elements of *MdANS*, *MdUFGT*, *MdANR*, and *MdDFR* genes to activate their expression in apple (Xie *et al.*, 2012; An *et al.*, 2015).

Anthocyanin biosynthesis is affected by multiple environmental stimuli such as intense light, UV irradiation, temperature, wounding, pathogen infection, nutrient deficiency, and drought, as well as by many endogenous developmental signals, such as sugar and plant hormones (Takos *et al.*, 2006; Lillo *et al.*, 2008; Jeong *et al.*, 2010; Li *et al.*, 2012; Sperdouli and Moustakas, 2012; Xie *et al.*, 2012; Zhang *et al.*, 2013; An *et al.*, 2015). Plant hormones such as auxins, jasmonic acid (JA), gibberellins (GAs), cytokinin (CKT), abscisic acid (ABA), and ethylene are involved in the modulation of anthocyanin biosynthesis (Mori *et al.*, 1994; Loreti *et al.*, 2008; Shan *et al.*, 2009; Jeong *et al.*, 2010). In poplar leaves, a variety of biotic and abiotic stresses such as nutrient deficiency, insect herbivory, pathogen attack, mechanical wounding, and intense light stimulate PA synthesis (Mellway *et al.*, 2009). JA also promotes PA accumulation by up-regulating the expression of the MYB genes *MdMYB9* and *MdMYB11* in apple (An *et al.*, 2015).

Sugar is very important throughout the entire plant life cycle because it acts as an energy source, a structural component, and an important regulatory molecule (Jang *et al.*, 1997). Sugar signaling generally regulates the TFs and the metabolic enzymes associated with pathogenesis, photosynthesis, nutrient mobilization and allocation, senescence, and anthocyanin biosynthesis at both the transcriptional and post-transcriptional levels (Koch, 1996; Buchanan-Wollaston *et al.*, 2005; Teng *et al.*, 2005; Rolland *et al.*, 2006). Sugars induce anthocyanin biosynthesis in Arabidopsis. The effect of sucrose and maltose is the most obvious in terms of elevating anthocyanin levels, followed by glucose, fructose, and turanose (a sucrose isomer). Other sugars such as galactose, lactose, and trehalose do not induce anthocyanin accumulation (Teng *et al.*, 2005; Góraj-Koniarska and Saniewski, 2015). The *MYB75/PAP1* gene is essential for sucrose-specific regulation of anthocyanin accumulation in Arabidopsis, which then enhances the transcriptional level of the anthocyanin biosynthetic genes (Solfanelli *et al.*, 2006). In addition, DELLA proteins are also found to be involved in sucrose-induced anthocyanin biosynthesis (Li *et al.*, 2014). However, the exact mechanism through which sucrose signaling controls the biosynthesis of anthocyanins is not yet clear.

Sugar-responsive pathways are highly complex processes involved in plant growth and development, and are integrated with other signaling pathways such as those for light, stresses, nitrogen, and plant hormones (Dijkwel *et al.*, 1997; Roitsch, 1999; Sun *et al.*, 2013; Ljung *et al.*, 2015). In higher plants, several components of sugar-responsive pathways have been identified by their conservation among eukaryotic cells (Jang *et al.*, 1997; Rolland *et al.*, 2006). HEXOKINASE1 (HXK1) and Sucrose-Nonfermenting1 (SNF1)-related protein kinases 1 (SnRK1) are the main players among them (Rolland *et al.*, 2006; Hanson and Smeekens, 2009). As the first plant sugar sensor, HXK1 senses glucose (Jang *et al.*, 1997; Moore *et al.*, 2003). In Arabidopsis, AtHXK1 has dual functions as a glycolytic enzyme and a sugar-responsive regulator which regulates gene expression and several plant hormone signaling transduction processes (Moore *et al.*, 2003). Similarly, two isoforms of the catalytic Arabidopsis SnRK1 α subunit, AtSnRK1.1 and AtSnRK1.2, are proposed to function as central integrators of transcription networks in response to stresses and sugar signaling (Baena-González *et al.*, 2007). They are structurally and functionally homologous to the yeast SNF1 and mammalian AMP-activated protein kinase (AMPK) (Baena-González *et al.*, 2007; Hardie, 2007). SnRK1 in plants is considered as a metabolic sensor that perceives the status of cellular carbohydrates and energy (Baena-González and Sheen, 2008; Ghillebert *et al.*, 2011).

SnRK1 is a heterotrimeric protein complex which is composed of a catalytic subunit α and two regulatory subunits, β and βγ within plants (Ramon *et al.*, 2013; Emanuelle *et al.*, 2016). The α subunit includes two functional domains: an N-terminal kinase domain (KD) that contains a conserved activation loop, and a C-terminal regulatory domain (RD) required for the interaction with β and βγ subunits (Crozet *et al.*, 2014; Emanuelle *et al.*, 2016). The β subunit acts as a scaffold to bridge α and βγ subunits (Crozet *et al.*, 2014). The βγ subunit, which functions as the canonical γ subunit, is required for SnRK1 complex formation in plants (Ramon *et al.*, 2013; Emanuelle *et al.*, 2016). In Arabidopsis, each subunit is encoded by multiple genes. As a result, there is a large variety of possible heterotrimeric combinations for SnRK1 kinase (Polge and Thomas, 2007; Emanuelle *et al.*, 2016).

SnRK1 is a Ser/Thr protein kinase, and it has been proved to phosphorylate and inactivate some important enzymes such as trehalose phosphate synthase 5 (TPS5), sucrose phosphate synthase (SPS), and nitrate reductase (NR) to modulate nutrient balance (Sugden *et al.*, 1999; Harthill *et al.*, 2006; Polge and Thomas, 2007). In addition to metabolic regulation, SnRK1 plays a crucial role in co-ordinating various stresses (Baena-González *et al.*, 2007). In plants, SnRK1 is important for seed filling, plant flowering, senescence, and maturation, as well as affecting both embryo and pollen development (Zhang *et al.*, 2001; Baena-González *et al.*, 2007; Radchuk *et al.*, 2010). It also responds to several phytohormones such as auxin, CKT, and ABA, and thus is involved in the crosstalk between sugar and hormone signaling pathways (Radchuk *et al.*, 2010). However, the exact mechanism underlying the potential link between SnRK1 and hormone signals is poorly understood.

Overexpression of *AtSnRK1.1* reduces anthocyanin accumulation under a sucrose concentration of 3% and represses the expression of *MYB75/PAP1* in Arabidopsis (Baena-González *et al.*, 2007). However, the exact molecular mechanism by which SnRK1 regulates anthocyanin biosynthesis remains to be elucidated. In this study, it was found that MdSnRK1.1, which is closely related to AtSnRK1.1, promoted the biosynthesis of anthocyanins and PAs in apple. Subsequently, its function in control of anthocyanin and PA biosynthesis by interacting with MdJAZ18 was characterized. Finally, the crosstalk between JA and sugar signaling was discussed.

## Materials and methods

### Plant materials and growth conditions

The calli of apple cultivar ‘Orin’ were subcultured at 20 d intervals on Murashige and Skoog (MS) medium containing 0.4 mg l^−1^ 6-benzylaminopurine (6-BA) and 1.5 mg l^−1^ 2,4-dichlorophenoxy acetic acid (2,4-D) at 25 °C in the dark. Shoot cultures of apple (*Malus domestica* ‘Royal Gala’) were subcultured at monthly intervals on MS medium supplemented with 0.5 mg l^−1^ 6-BA and 0.2 mg l^−1^ naphthylacetic acid (NAA) at 25 °C under long-day conditions.

*Arabidopsis thaliana* ecotype ‘Columbia’ was used as the background for genetic transformation and control. After vernalization, seeds were sown on MS medium without sucrose for 4 d, and then treated with different sucrose concentrations (0, 1, 3, 6, and 9%) under long-day conditions for 1 week.

To analyze the accumulation of anthocyanin and PA, apple calli in good condition were transferred to the calli culture medium with low nitrogen (0.5 mM) and different concentrations of sucrose (1, 3, 6, 9, and 12%) and mannitol (1, 3, and 6%) in the dark for 2 d, and then were exposed to 17 °C with continuous UVB (280–320 nm) light for 1 week. The *in vitro* apple shoot cultures were treated at 17 °C under continuous white light (30 mmol m^−2^ s ^−1^) for 2 weeks after starvation.

### Anthocyanin measurement

Anthocyanins were extracted from the samples with a HCl–methanol method, and the content was calculated following the protocol described by An *et al.* (2015). The experiment was repeated at least three times for each sample.

### PA extraction and determination

The DMACA (*p*-dimethylaminocinnamaldehyde)–methanol method was used to stain PAs in apple calli and leaves. PAs were extracted from the samples by the method described previously (An *et al.*, 2015). (+)-Catechin hydrate was used to check the quantity. The experiment was repeated at least three times for each sample.

### RNA extraction and quantitative real-time PCR (qRT-PCR) analysis

The *in vitro* apple shoot cultures and calli were used for RNA extraction. RNAs were extracted from the *in vitro* apple shoot cultures and calli using RNAplant Plus Reagent (Tiangen, Beijing, China), and then reverse transcribed using a PrimeScript first-strand cDNA synthesis kit (Takara, Dalian, China), following the manufacturer’s instructions. qRT-PCR assays were performed with the UltraSYBR Mixture (SYBR Green I) (Takara) using an ABI7500 qRT-PCR system. The concentration of cDNA was diluted to 1–10 ng μl^−1^. A 1 μl aliquot of diluted cDNA was used for qRT-PCR. The calculation method for qRT-PCR is 2^–ΔΔ^CT. The results were normalized by *18S*. At least three replicates for each sample were used for qRT-PCR. The primers used are listed in Supplementary Table S1 at *JXB* online or in An *et al.* (2015).

### Vector constructs and plant transformation

To construct the expression vectors, the full-length cDNA of *MdJAZ18* and *MdSnRK1.1* genes, and an antisense fragment (asMdSnRK1.1) specific to the *MdSnRK1.1* gene were cloned from apple ‘Royal Gala’.

*MdSnRK1.1* cDNA was linked with PXSN-MYC (Chen *et al.*, 2009) and PRI–green fluorescent protein (GFP), respectively; the antisense fragment asMdSnRK1.1 was linked with PXSN and PRI, respectively; while *MdJAZ18* cDNA was linked with PXSN-FLAG and PRI–β-glucosidase (GUS), respectively. These vectors were driven by the *Cauliflower mosaic virus* (CaMV) 35S promoter. The primers used are shown in Supplementary Table S1.

The recombinant plasmids were transferred into *Agrobacterium tumefaciens* strain LBA4404, and then introduced into apple calli using the method described by An *et al.* (2012). For Arabidopsis, the ecotype ‘Columbia’ was used for transformation with an *Agrobacterium tumefaciens* GV3101-mediated floral dip method. Transgenic lines were screened with kanamycin monosulfate. Homozygous transgenic lines were used in the experiment.

### Yeast two-hybrid (Y2H) screening and Y2H assays

Full-length cDNA of the *MdSnRK1.1* gene was cloned into the bait vector pGBT9. Y2H screening of the apple cDNA library was performed according to the yeast transformation system (Clontech, Dalian, China), and screened on yeast dropout medium lacking Trp, Leu, His, and Ade (-T/-L/-H/-A).

Y2H assays were performed as described in the manufacturer’s instructions (Clontech). The domain fragments of the *MdSnRK1.1* gene were each cloned into vector pGBT9, while those of the full-length *MdJAZ18* gene were cloned into vector pGAD424. To confirm the general interaction in plants, *AtSnRK1.1* was cloned into pGBT9, and *AtJAZ3*, *MdJAZ1*, *MdJAZ8*, *MdJAZ9*, *MdJAZ10*, *MdJAZ12*, *MdJAZ14*, *MdJAZ18*, and *MdJAZ19* were cloned into pGAD424. Primers used for vector construction are shown in Supplementary Table S1.

### Pull-down assays

The coding regions of *MdSnRK1.1* and *MdJAZ18* were introduced into the PET-32a and PGEX-4T-1 vector, respectively, and then recombinant vector was transformed into *Escherichia coli* BL21(DE3) to express HIS-MdSnRK1.1 or GST–MdJAZ18 protein. The pull-down assay was performed according to the instructions of the Pierce GST Spin Purification Kit (Thermo, MA, USA). Then samples were detected by immunoblotting with anti-GST and anti-HIS antibodies, respectively.

### In vitro *kinase assays*

*In vitro* kinase assays were implemented according to the method described by Lin *et al.* (2009). For *in vitro* kinase assays, the *E. coli* strain BL21-induced HIS-MdSnRK1.1, HIS-MdJAZ18, and HIS-MdJAZ12 fused proteins were purified and co-incubated in kinase buffer (20 mM Tris, pH 7.5, 20 mM DTT, 50 mM MgCl_2_, 100 μM ATP, and 100 mM MnCl_2_) containing 10 μCi of [γ-^32^P]ATP at 30 °C for 30 min. Proteins were separated by SDS–PAGE, and phosphorylated protein was detected by exposing the dried gels to X-ray films.

### *Protein extraction and protein degradation* in vitro

Dry apple calli were harvested and powdered in liquid nitrogen. The residues were mixed with degradation buffer [25 mM Tris–HCl, pH 7.5, 10 mM NaCl, 10 mM MgCl_2_, 4 mM phenylmethylsulfonylfluoride (PMSF), 5 mM DTT, and 10 mM ATP]. The mixture was kept for 15 min on ice, and then centrifuged for 15 min at 12 000 *g* at 4 °C. The supernatant was collected, and the Bradford assay was used to detecte its protein concentration. MdACTIN was used as internal non-degraded control. A concentration of 100 μM MG132 (carbobenzoxy-l-leucyl-l-leucyl-l-leucinal), which is proteasome inhibitor, was added to the degradation system as indicated. For protein degradation *in vitro*, 100 ng of *E. coli* strain BL21-induced recombinant HIS-MdJAZ18 protein was incubated in 600 μl extracts (containing 500 µg of total proteins) for each reaction system at 22 °C for the indicated times. The HIS-MdJAZ18 protein abundance was detected by immunoblotting with an anti-HIS antibody, and quantified with Bio-Rad’s QuantityOne software.

### Transient expression and GUS analysis assays

The transient expression assays were performed in apple calli. The coding sequence of *MdSnRK1.1* was cloned into the virus plasmid vector pIR, and IL-60-BS functioned as an auxiliary construct for the normal replication, movement, and expression of pIR (Peretz *et al.*, 2007). About 2 μg ml^−1^ of plasmid was introduced into wild-type (WT) and MdJAZ18–GUS calli, while JA and calf intestinal alkaline phosphatase (CIP) were selectively added to various reaction systems as indicated. Thereafter, the calli were placed under normal conditions for 4 d. For GUS histochemical staining, the same quality of dry calli was immersed in GUS staining buffer {0.5 μg μl^−1^ X-Gluc, 0.075 M sodium phosphate buffer pH 7.2, 0.05 mM K_4_[Fe(CN)]_6_·3(H_2_O), 0.05 mM K_3_[Fe(CN)_6_], 10 mM EDTA, 20% methanol, 0.1% Triton X-100}. The samples were incubated overnight at 37 °C.

To detect the GUS activity, 500 mg of calli were ground and extracted with 1 ml of GUS extraction buffer (50 mM sodium phosphate buffer pH 7.0, 10 mM EDTA, 0.1% Triton X-100, 0.1% *N*-lauroylsarcosine). The GUS activity assay was performed as described by An *et al.* (2015). The analysis was repeated at least three times.

## Results

### Sucrose induces anthocyanin and PA accumulation in apple

**Fig. 1.**
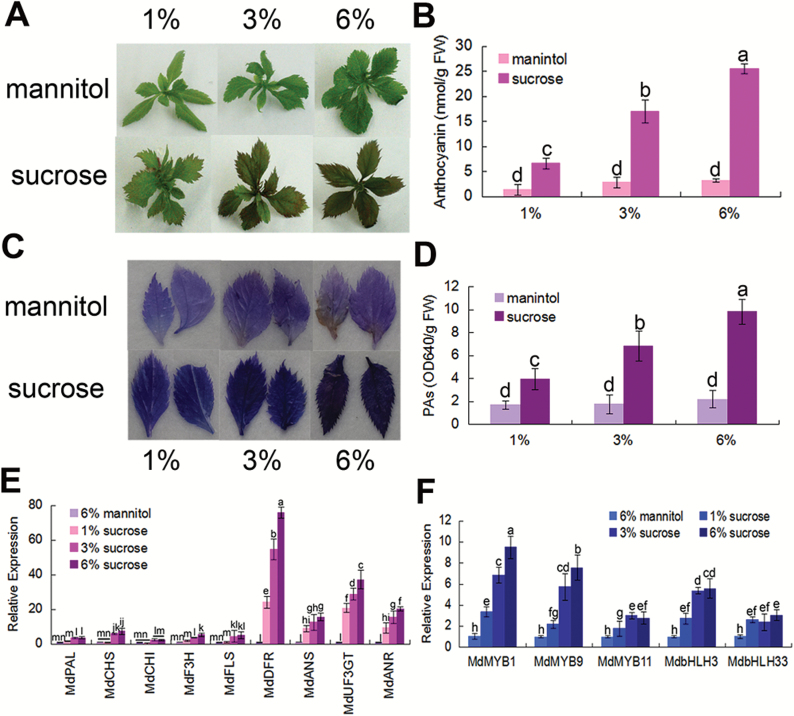
Sucrose induces anthocyanin and PA accumulation by promoting the expression of the regulatory and biosynthetic genes. (A, B) Anthocyanin pigmentation phenotype (A) and anthocyanin contents (B) of 20-day-old *in vitro* apple shoot cultures treated with different concentrations (1, 3, and 6%) of sucrose and mannitol. (C, D) PA staining and content determination in apple leaves of the shoot cultures in (A). In (B) and (D), FW, fresh weight. Error bars represent the SDs, which were analyzed based on >9 replicates. (E, F) Expression analysis of anthocyanin- and PA-related genes with qRT-PCR in the plants shown in (A). *18S* was used as the internal control. Error bars represent the SD based on three independent replicates. In (B), (D), (E), and (F), statistical significance was calculated by the LSD test with DPS software, *P*<0.05.

To examine if sucrose promotes anthocyanin and PA accumulation in apple, *in vitro* shoot cultures of apple grown on MS medium containing sucrose were used. The results showed that anthocyanin levels increased with sucrose, but not with the osmotic control mannitol (Fig. 1A, B). Similarly, sucrose also promoted the accumulation of PAs in the apple leaves (Fig. 1C, D). Furthermore, the expression levels of genes involved in anthocyanin and PA biosynthesis were examined by qRT-PCR assays. When compared with mannitol, the results indicated that sucrose noticeably promoted the transcript levels of anthocyanin and PA structural genes such as *MdUF3GT*, *MdANR*, *MdANS*, and *MdDFR*, while the transcript levels of *MdPAL*, *MdCHS*, *MdF3H*, *MdCHI*, and *MdFLS* increased a little (Fig. 1E). The expression levels of the regulatory MYB and bHLH genes were also assayed. The results indicated that the mRNA levels of *MdMYB1*, *MdMYB9*, *MdMYB11*, *MdbHLH3*, and *MdbHLH33* were also noticeably enhanced in response to sucrose treatment (Fig. 1F).

### MdSnRK1.1 *is involved in sucrose-induced accumulation of anthocyanins and PAs*

SnRK1 plays a crucial role in sugar and metabolic signaling pathways in plants (Baena-González *et al.*, 2007; Mohannath *et al.*, 2014). To characterize the function of SnRK1 in anthocyanin and PA accumulation in response to sucrose, three *AtSnRK1*-like genes, MDP0000191788 (*MdSnRK1.1*), MDP0000173500 (*MdSnRK1.2*), and MDP0000320932 (*MdSnRK1.3*) were found in apple. They are similar to each other (Supplementary Fig. S1A). The Neighbor–Joining phylogenetic tree based on the amino acid sequences of AtSnRK1s and MdSnRK1s showed that MdSnRK1.1 had the highest similarity to AtSnRK1.1 (Supplementary Fig. S2). The *MdSnRK1.1* gene was cloned from apple (Li *et al.*, 2010). The alignment analysis of the amino acid sequences demonstrated that the predicted MdSnRK1.1 protein shares 82.16% similarity with the AtSnRK1.1 protein in Arabidopsis. It contains a conserved KD and a C-terminal RD (Supplementary Fig. S1B).

To characterize the function of the *MdSnRK1.1* gene, the calli of apple cultivar ‘Orin’ were used for genetic transformation. The full-length cDNAs of the *MdSnRK1.1* gene were used to construct the overexpression vector 35S::MdSnRK1.1-GFP, while its antisense cDNA fragment was used to construct the suppression vector 35S::asMdSnRK1.1. As a result, two transgenic calli, 35S::MdSnRK1.1-GFP and 35S::asMdSnRK1.1, were obtained. The expression analysis demonstrated that 35S::MdSnRK1.1-GFP transgenic calli produced much more *MdSnRK1.1* transcript, but 35S::asMdSnRK1.1 calli produced less compared with the WT control (Suupplementary Fig. S3A), thereby indicating that the expression of the *MdSnRK1.1* gene was successfully overexpressed or suppressed in the transgenic calli. Moreover, the expression of *MdSnRK1.2* and *MdSnRK1.3* was also reduced in 35S::asMdSnRK1.1 transgenic calli, perhaps due to the sequence similarity of their products to MdSnRK1.1 (Supplementary Fig. S3B).

**Fig. 2.**
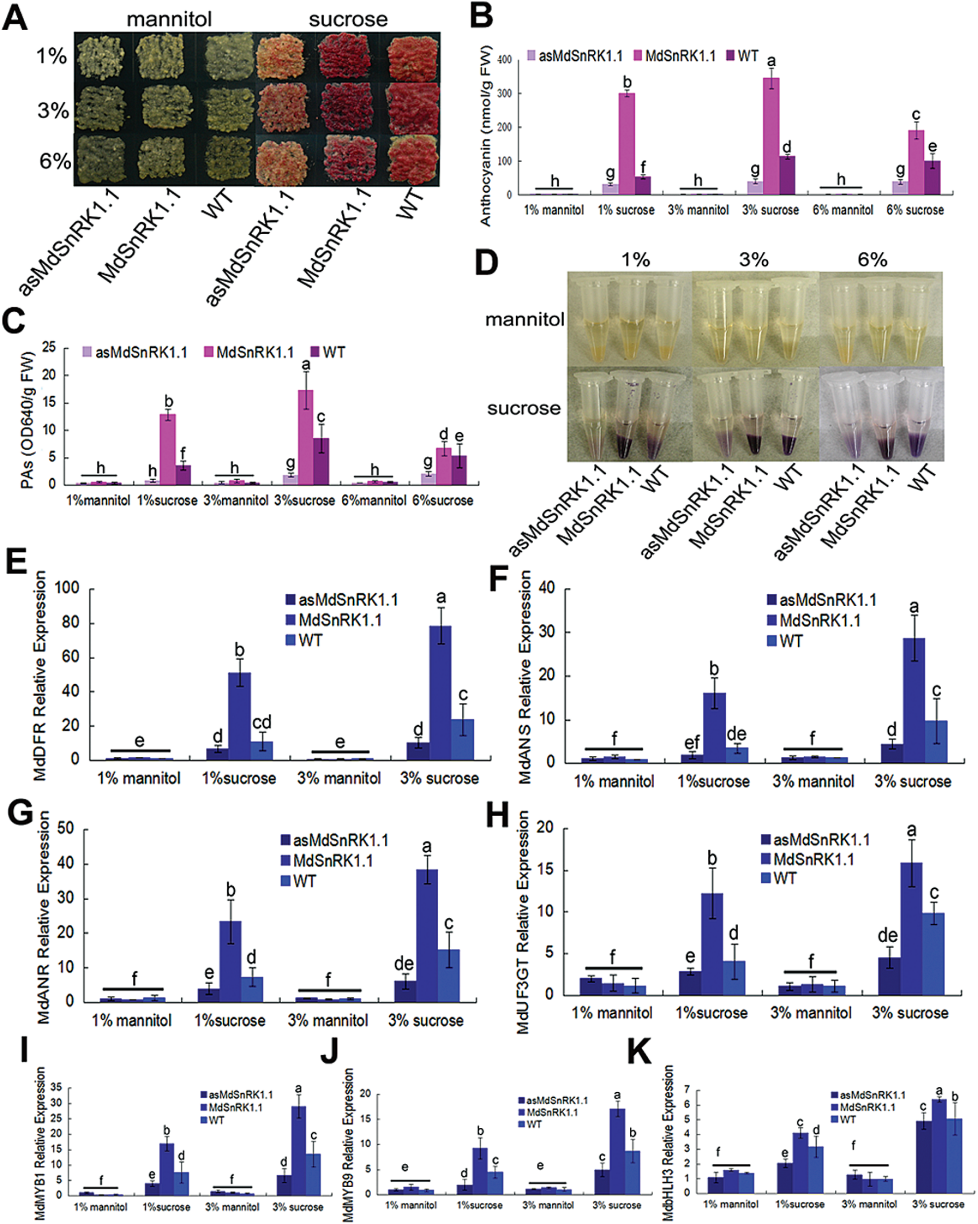
*MdSnRK1.1* enhances anthocyanin and PA accumulation. (A) Coloration of the WT apple calli and the transgenic apple calli (*MdSnRK1.1* and asMdSnRK1.1) treated with sucrose (1, 3, and 6%) or mannitol (1, 3, and 6%). (B–D) Anthocyanin contents (B), PA contents (C), and staining (D) in the corresponding calli shown in (A). In (B) and (C), FW, fresh weight. (E–K) The expression levels of the regulatory *MdbHLH3*, *MdMYB1*, and *MdMYB9* genes, as well as the structural *MdANS*, *MdDFR*, *MdUF3GT*, and *MdANR* genes in anthocyanin and PA biosynthetic pathways in the calli shown in (A), as analyzed with qRT-PCR. *18S* was used as the internal control. In (B), (C). and (E–K), error bars represent the SD based on three independent replicates. Statistical significance was calculated by the LSD test with DPS software, *P*<0.05.

Subsequently, two kinds of transgenic calli were used to examine whether MdSnRK1.1 influences the accumulation of anthocyanins and PAs in response to sucrose, and WT calli were used as the control. In the absence of sucrose, three kinds of calli failed to produce anthocyanins on the medium with mannitol, thus indicating that sugar is necessary for their accumulation. When exposed to different concentrations of sucrose, the WT control produced more anthocyanins in response to 3% sucrose than to 1% and 6% sucrose (Fig. 2A, B).

For the transgenic calli, the overexpression of the *MdSnRK1.1* gene noticeably increased the accumulation of anthocyanins under 1% sucrose. The 35S::MdSnRK1.1-GFP transgenic calli accumulated more anthocyanins under 1% sucrose, even more than the WT control under the most appropriate sucrose (3%) condition (Fig. 2A, B). However, high sucrose (>3% in the case of apple calli) reduced the accumulation of anthocyanins, compared with 1% sucrose (Supplementary Fig. S4A, B). In contrast, the suppression of *MdSnRK1.1* neutralized sucrose-induced anthocyanin accumulation. The 35S::asMdSnRK1.1 transgenic calli produced anthocyanins at similar levels under the different tested sucrose concentrations (Fig. 2A, B), indicating that MdSnRK1.1 is necessary for the sucrose-induced anthocyanin accumulation. In addition, the accumulation of PAs was also detected. The result showed that PAs accumulated in the calli in a pattern highly similar to that of anthocyanins in response to the tested sucrose concentrations (Fig. 2C, D).

Furthermore, the expression of flavonoid biosynthetic genes associated with anthocyanin and PA accumulation was analyzed with qRT-PCRs in WT control as well as 35S::MdSnRK1.1-GFP and 35S::asMdSnRK1.1 transgenic calli treated with different concentrations of sucrose. Corresponding to the anthocyanin and PA phenotypes, the expression levels of LBGs such as *MdUF3GT*, *MdDFR*, *MdANS*, and *MdANR* were significantly up-regulated in 35S::MdSnRK1.1-GFP transgenic calli (Fig. 2E–H). Besides these structural genes, MdSnRK1.1 also positively regulated the expression of anthocyanin-related regulatory upstream genes such as *MdMYB1* and *MdMYB9*, but only slightly influenced the expression of the *MdbHLH3* gene (Fig. 2I–K). In contrast, the suppression of the *MdSnRK1.1* gene neutralized the sucrose-induced expression in 35S::asMdSnRK1.1 transgenic calli as compared with the WT control (Fig. 2E–K).

In addition, the expression vector 35S::MdSnRK1.1-GFP was genetically transformed into Arabidopsis to verify the involvement of MdSnRK1.1 in the regulation of anthocyanin biosynthesis (Supplementary Fig. S3C). It was found that *MdSnRK1.1* transgenic lines exhibited sucrose-hypersensitive phenotypes. In these *MdSnRK1.1* transgenic lines, 1% sucrose promoted root growth and anthocyanin accumulation, while 6% sucrose inhibited them (Supplementary Figs S4C, D, S5).

### MdSnRK1.1 interacts with MdJAZ18 protein

**Fig. 3.**
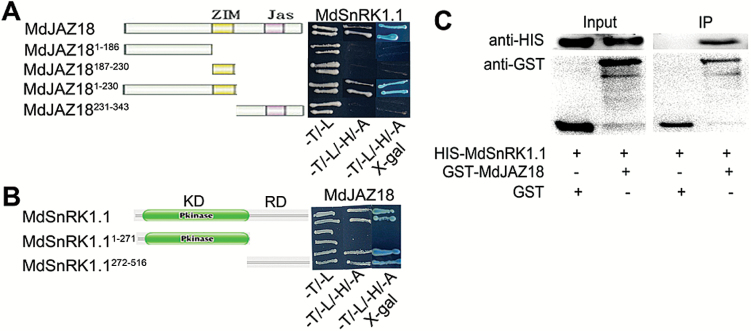
MdSnRK1.1 interacts with MdJAZ18. (A, B) MdSnRK1.1 interacts with MdJAZ18 in Y2H assays. According to the domains of MdSnRK1.1 (KD and RD domain) and MdJAZ18 (ZIM and Jas domain), the full length or derivatives thereof were used to assess their interactions. Yeast dropout medium lacking Leu and Trp (-T/-L) was used as transformation control, and that lacking Leu, Trp, His, and Ade (-T/-L/-H/-A) acted as a screen. (C) *In vitro* pull-down assay to verify the interaction between MdSnRK1.1 and MdJAZ18. HIS-MdSnRK1.1 was co-incubated with GST or GST–MdJAZ18 protein and then purified using a GST purification kit. The resultant protein samples were immunoblotted with anti-HIS and anti-GST antibodies, respectively.

To elucidate how MdSnRK1.1 modulates the accumulation of anthocyanins and PAs, Y2H screening through a cDNA library was performed. The full-length cDNA of the *MdSnRK1.1* gene was inserted into the pGBT9 vector. The resultant vector BD-MdSnRK1.1 was used as a bait to screen the library for MdSnRK1.1-interacting proteins. The result showed that a positive colony contained a cDNA fragment which is a part of the *MdJAZ18* gene (Li *et al.*, 2015). The evidence suggests that MdJAZ18 plays an important role in the regulation of anthocyanin and PA biosynthesis (An *et al.*, 2015). Therefore, the Y2H assay was conducted to verify the interaction. The full-length cDNA of the *MdJAZ18* gene was inserted into the pGAD424 vector as prey (AD-MdJAZ18). The different combinations of bait and prey vectors as well as empty control were transformed into yeast for Y2H assays. The result showed that MdSnRK1.1 interacted with MdJAZ18 protein (Fig. 3A). Furthermore, the interactions between MdSnRK1.1 and other MdJAZs such as MdJAZ1, MdJAZ8, MdJAZ9, MdJAZ10, MdJAZ12, MdJAZ14, and MdJAZ19, were also assayed (Supplementary Fig. S6). The result indicated that MdSnRK1.1 interacted only with MdJAZ18, but not with the other proteins (Supplementary Fig. S7A). In addition, Arabidopsis AtJAZ3 is the orthologous protein of MdJAZ18. The Y2H assay demonstrated that AtJAZ3 also interacted with both MdSnRK1.1 and AtSnRK1.1 proteins (Supplementary Fig. S7A, B).

To map the domains in MdSnRK1.1 and MdJAZ18 proteins necessary for their interaction, MdSnRK1.1 was divided into an N-terminal kinase domain MdSnRK1.1^1–271^ and a C-terminal regulatory domain MdSnRK1.1^272–516^, while MdJAZ18 was divided into MdJAZ18^1–186^, MdJAZ18^187–230^, MdJAZ18^1–230^, and MdJAZ18^231–343^. They were then inserted into pGBT9 and pGAD424 vectors, respectively. As a result, six vectors BD-MdSnRK1.1^1–271^, BD-MdSnRK1.1^272–516^, AD-MdJAZ18^1–186^, AD-MdJAZ18^187–230^, AD-MdJAZ18^1–230^, and AD-MdJAZ18^230–343^ were obtained and used for Y2H assays. The results showed that C-terminal regulatory domain (272–516) of MdSnRK1.1 protein and the N-terminal ZIM domain (1–230) of MdJAZ18 protein were responsible for their interaction (Fig. 3A, B).

To verify further the interaction between MdSnRK1.1 and MdJAZ18, an *in vitro* pull-down assay was conducted. Purified recombinant HIS-MdSnRK1.1 and GST–MdJAZ18 proteins were expressed and purified from *E. colli* BL21. Subsequently, HIS-MdSnRK1.1 proteins were incubated with GST–MdJAZ18 and GST, respectively, and then separated by SDS–PAGE for western blotting with an anti-HIS antibody. The HIS-MdSnRK1.1 proteins were enriched by GST–MdJAZ18, but not by the GST control (Fig. 3C), indicating that MdSnRK1.1 physically interacted with MdJAZ18.

### MdSnRK1.1 phosphorylates and destabilizes MdJAZ18 protein

**Fig. 4.**
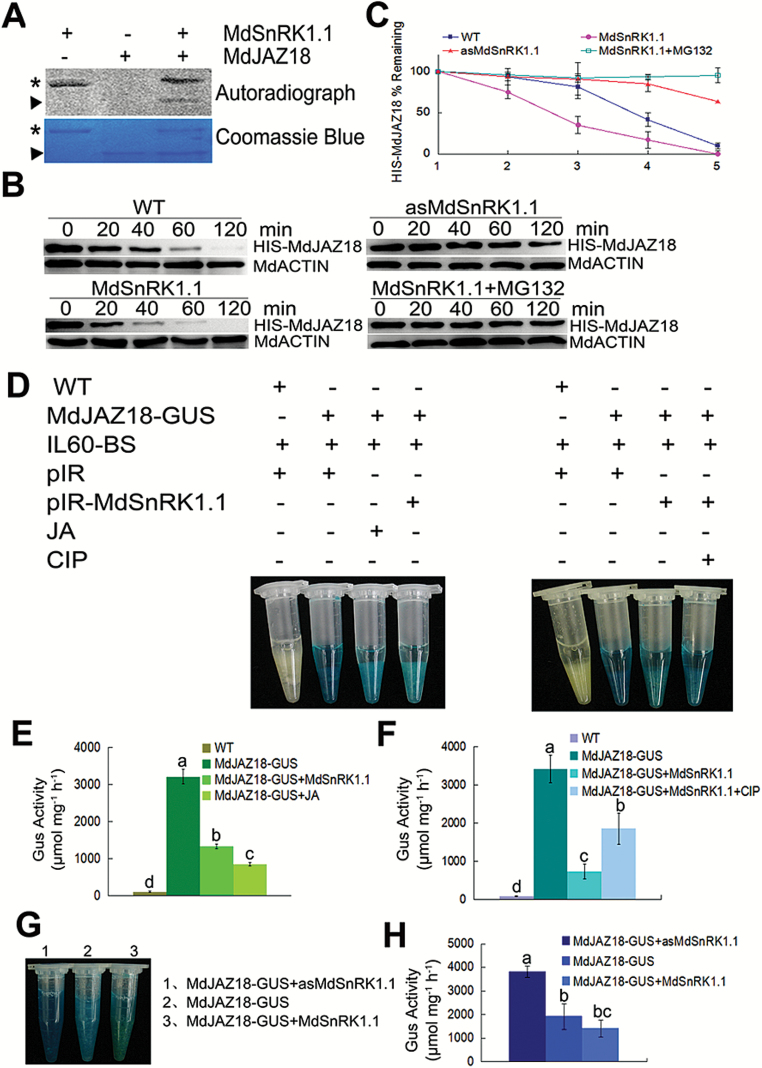
MdSnRK1.1 phosphorylates and degrades MdJAZ18 protein. (A) MdSnRK1.1 phosphorylates MdJAZ18 *in vitro*. The asterisk indicates the autophosphorylation of the purified HIS-MdSnRK1.1, while the triangle indicates the phosphorylation of the purified HIS-MdJAZ18 by HIS-MdSnRK1.1 in the autoradiogram. The asterisk and the triangle refer to protein loading of HIS-MdJAZ18 and HIS-MdSnRK1.1, respectively, in Coomassie blue staining. (B) MdJAZ18 stability is reduced by MdSnRK1.1 and the effect is inhibited by MG132. Total proteins extracted from WT, MdSnRK1.1, and asMdSnRK1.1 apple calli were incubated with the purified HIS-MdJAZ18 protein treated with or without MG132. The samples were harvested at the indicated time. MdACTIN was used as internal reference. (C) The half-life plot for *in vitro* HIS-MdJAZ18 degradation was analyzed from protein bands with Bio-Rad QuantityOne software. (D) MdSnRK1.1 degrades MdJAZ18, and CIP inhibits MdSnRK1.1-mediated degradation. GUS staining images of transiently expressed pIR-MdSnRK1.1 or pIR in MdJAZ18-GUS calli treated with or without JA or CIP. WT calli were used as a negative control. (E, F) Quantitative analysis of GUS activity in (D). (G) MdSnRK1.1 promoted the degradation of MdJAZ18 *in vivo*. GUS staining images of MdJAZ18–GUS+MdSnRK1.1, MdJAZ18–GUS+asMdSnRK1.1, and MdJAZ18–GUS calli. (H) Quantitative analysis of GUS activity in (G). In (C), (E), (F), and (H), error bars represent the SD. Statistical significance was calculated by the LSD test with DPS software, *P*<0.05.

MdSnRK1.1 is a Ser/Thr protein kinase. To examine whether MdSnRK1.1 phosphorylates MdJAZ18 protein, the purified HIS-MdSnRK1.1, HIS-MdJAZ18, and HIS-MdJAZ12 proteins were used for an *in vitro* phosphorylation experiment. The result showed that MdSnRK1.1 efficiently autophosphorylated itself, indicating its kinase activity. The phosphorylated MdJAZ18 protein was detected in the MdSnRK1.1 and MdJAZ18 co-incubated sample, but not in the MdJAZ18 sample alone, indicating that MdSnRK1.1 directly phosphorylated MdJAZ18 protein *in vitro* (Fig. 4A). In addition, MdSnRK1.1 failed to phosphorylate MdJAZ12 protein (Supplementary Fig. S8).

JAZs are typical proteins which interact with the SCF^COI1^ complex and are degraded via the ubiquitination–26S proteasome pathway (Thines *et al.*, 2007). To examine if MdSnRK1.1-mediated phosphorylation influences the stability of MdJAZ18 protein, a cell-free degradation assay was carried out. The prokaryon-expressed and purified HIS-MdJAZ18 protein was incubated with the plant total proteins that were extracted from WT control, 35S::MdSnRK1.1-GFP, and 35S::asMdSnRK1.1 transgenic apple calli. Subsequently, immunoblottings were performed with an anti-HIS antibody to detect the protein abundance. The results showed that the abundance of HIS-MdJAZ18 protein was less in the protein extracts of 35S::MdSnRK1.1-GFP transgenic calli than in those of the WT control (Fig. 4B, C), whereas it was more stable in the protein extracts of 35S::asMdSnRK1.1 transgenic calli than in those of the WT control (Fig. 4B, C). In addition, MdSnRK1.1-mediated HIS-MdJAZ18 degradation was more rapid in protein samples of 35S::MdSnRK1.1-GFP transgenic calli treated with 1% sucrose than those treated with 3% and 6% sucrose (Supplementary Fig. S9). These results suggest that MdSnRK1.1-mediated phosphorylation of MdJAZ18 protein promotes its degradation in response to sucrose. Furthermore, the MG132 treatment inhibited MdSnRK1.1-mediated degradation of MdJAZ18 protein (Fig. 4B, C), indicating that the degradation may be through a 26S proteasome pathway.

To detect the degradation of MdJAZ18 *in vivo*, the 35S::MdJAZ18-GUS construct was genetically transformed into apple calli. The GUS staining assay demonstrated that 35S::MdJAZ18-GUS transgenic calli exhibited GUS activity, indicating that MdJAZ18–GUS fusion protein was successfully expressed in the transgenic calli. After treatment with 100 μM JA for 2 h, the transgenic calli showed a reduced GUS activity, indicating that MdJAZ18 proteins were degraded in response to JA (Supplementary Fig. S10).

To check rapidly if MdSnRK1.1 influences the degradation of MdJAZ18, the viral vectors pIR and pIR-MdSnRK1.1 were used to transiently transform 35S::MdJAZ18-GUS transgenic calli. The GUS staining assay demonstrated that 35S::MdJAZ18-GUS+pIR-MdSnRK1.1 double transgenic calli showed a lower GUS activity than the 35S::MdJAZ18-GUS+pIR control, indicating that MdSnRK1.1 promoted the degradation of MdJAZ18 protein (Fig. 4D, E). However, CIP treatment partially neutralized MdSnRK1.1-mediated degradation of MdJAZ18–GUS protein (Fig. 4D, F), suggesting that phosphorylation modification was involved in the degradation process.

To verify further that MdSnRK1.1 promotes MdJAZ18 degradation, double transgenic calli 35S::MdJAZ18-GUS+35S:: MYC-MdSnRK1.1 and 35S::MdJAZ18-GUS+35S::asMdSn RK1.1 were obtained (Supplementary Fig. S11A, B). Subsequently, they were used to detect the abundance of MdJAZ18–GUS protein with a GUS staining assay, while 35S::MdJAZ18-GUS transgenic calli were used as the control. The results showed that 35S::MdJAZ18-GUS+35S:: MYC-MdSnRK1.1 double transgenic calli exhibited much lower GUS activity, while that in 35S::MdJAZ18-GUS+35S:: asMdSnRK1.1 calli was higher than in the 35S::MdJAZ18-GUS control (Fig. 4G, H).

Taken together, these findings indicate that MdSnRK1.1 phosphorylates MdJAZ18 to facilitate its 26S proteasome-mediated degradation.

### MdJAZ18 is involved in MdSnRK1.1-induced anthocyanin and PA accumulation

**Fig. 5.**
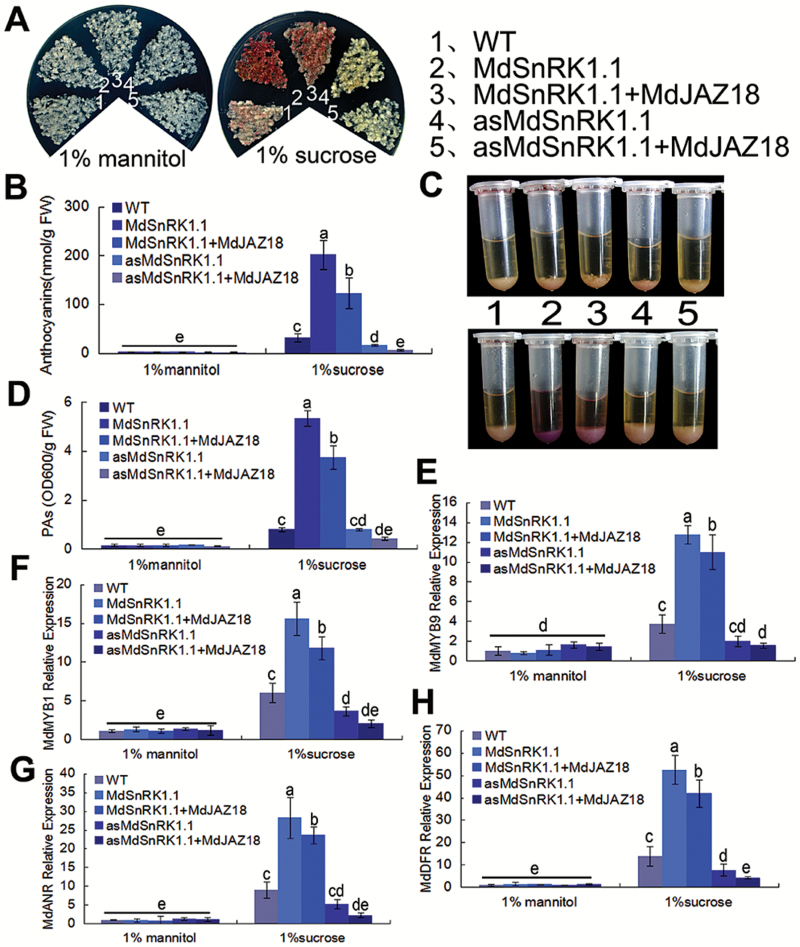
MdJAZ18 is involved in MdSnRK1.1-induced anthocyanin and PA accumulation. (A, B) Coloration (A) and anthocyanin contents (B) of WT, 35S::MdSnRK1.1-GFP, 35S::MdSnRK1.1-GFP+35S::Flag-MdJAZ18, 35S::asMdSnRK1.1, and 35S::asMdSnRK1.1 + 35S::Flag-MdJAZ18 treated with 1% sucrose or mannitol. (C, D) PA staining (C) and contents (D) in the corresponding calli shown in (A). In (B) and (D), FW, fresh weight. (E–H) The expression levels of regulatory genes (*MdMYB1* and *MdMYB9*) and structural genes (*MdDFR* and *MdANR*) in anthocyanin and PA biosynthetic pathways by qRT-PCR analysis in the calli shown in (A). *18S* acted as the internal control. In (B) and (D–H), error bars represent the SD based on three independent replicates. Statistical significance was calculated by the LSD test with DPS software, *P*<0.05.

In plants, JAZ proteins degrade in response to JA signal, which release bHLH and MYB TFs to promote anthocyanin and PA biosynthesis (Qi *et al.*, 2011; An *et al.*, 2015). To examine if MdJAZ18 functions in MdSnRK1.1-mediated anthocyanin and PA accumulation, the fused Flag-MdJAZ18 driven by a *35S* promoter was genetically transformed into WT calli, as well as into 35S::MdSnRK1.1-GFP and 35S::asMdSnRK1.1 transgenic calli. The resultant 35S::MdSnRK1.1-GFP, 35S::asMdSnRK1.1, 35S::MdSnRK1.1-GFP+35S::Flag-MdJAZ18, and 35S::asMdSnRK1.1 + 35S::Flag-MdJAZ18 transgenic calli were obtained and used for anthocyanin and PA analysis (Supplementaty Fig. S11A), while WT calli were applied as the control. The results showed that sucrose is necessary for anthocyanin and PA biosynthesis in all tested calli, and that MdSnRK1.1 promoted anthocyanin and PA accumulation in response to sucrose (Fig. 5A–D).

When *MdJAZ18* was overexpressed in 35S::MdSnRK1.1-GFP transgenic calli, 35S::MdSnRK1.1-GFP+35S::Flag-MdJAZ18 double transgenic calli produced less anthocyanins and PAs than 35S::MdSnRK1.1-GFP, indicating that Flag-MdJAZ18 oversupply neutralized MdSnRK1.1-mediated MdJAZ18 degradation, thereby inhibiting MdSnRK1.1-promoted anthocyanin and PA accumulation (Fig. 5A–D). In addition, it was found that the suppression of the *MdSnRK1.1* gene inhibited sucrose-induced anthocyanin and PA accumulation in 35S::asMdSnRK1.1 transgenic calli, and that the *MdJAZ18* overexpression exacerbated this inhibition in 35S::antiMdSnRK1.1 + 35S::Flag-MdJAZ18 calli (Fig. 5A–D). These findings indicated that MdJAZ18 was involved in MdSnRK1.1-mediated anthocyanin and PA accumulation in response to sucrose.

Consistent with the anthocyanin and PA phenotypes, the expression levels of the regulatory MYB genes *MdMYB1* and *MdMYB9*, as well as the structural genes *MdDFR*, *MdANS*, *MdANR*, and *MdUF3GT*, were lower in 35S::MdSnRK1.1-GFP+35S::Flag-MdJAZ18 and 35S::asMdSnRK1.1 + 35S::Flag-MdJAZ18 transgenic calli than in the corresponding 35S::MdSnRK1.1-GFP and 35S::asMdSnRK1.1 calli (Fig. 5E–H: Supplementary Fig. S12A, B). These findings suggested that MdJAZ18 was involved in MdSnRK1.1-induced anthocyanin and PA accumulation by modulating the expression of the regulatory and structural genes.

## Discussion

The stimulatory effects of sucrose on anthocyanin biosynthesis have been reported in different plant species such as grapevine, petunia, mulberry, and Arabidopsis (Pirie and Mullins, 1976; Weiss, 2000; Teng *et al.*, 2005; Tsai *et al.*, 2005). The sucrose-induced anthocyanin biosynthesis depends on the MYB75/PAP1 TF, but not on the glucose sensor AtHXK1 in Arabidopsis (Teng *et al.*, 2005). Signaling intermediates such as Ca^2+^, protein kinases, and protein phosphatases participate in sucrose-induced anthocyanin accumulation (Vitrac *et al.*, 2000). In parallel, various stresses and phytohormones induce the accumulation of PAs by modulating the expression of their biosynthetic genes (Mellway *et al.*, 2009; An *et al.*, 2015; Yoshida *et al.*, 2015). In this study, it was found that sucrose induced the biosynthesis of anthocyanins and PAs in apple, and that MdSnRK1.1 played a crucial role by interacting with and phosphorylating MdJAZ18 protein in this process (Figs 1A–D, 2A–D, 3A–C, 4A).

SnRK1 is an important regulator of plant growth and development (Baena-González *et al.*, 2007; Lu *et al.*, 2007; Cho *et al.*, 2012). It positively regulates the accumulation of starch in potato tubers (Lin *et al.*, 2014). It also inhibits seed germination and seedling growth by positively regulating the expression of *MYBS1* and *aAmy3* genes in rice (Lu *et al.*, 2007). In addition, sugar is indispensible for anthocyanin accumulation. Both sugar starvation and oversupply inhibit anthocyanin biosynthesis (Sivitz *et al.*, 2008). SnRK1 is proposed to function as a central component of kinase cascades in the sugar signaling pathway (Baena-González *et al.*, 2007; Jossier *et al.*, 2009). Its activity is regulated by sugar (Baena-González and Sheen, 2008).

Correspondingly, *SnRK1.1* overexpression increases the sensitivity to sucrose both in Arabidopsis and in apple calli (Jossier *et al.*, 2009; Fig. 2A; Supplementary Fig. S5). In this case, 3% sucrose is appropriate for the WT Arabidopsis plants, but is too high for the transgenic plants. Therefore, 3% sucrose inhibited anthocyanin accumulation in *AtSnRK1.1* transgenic Arabidopsis (Baena-González *et al.*, 2007). In this study, ectopic expression of the *MdSnRK1.1* gene in Arabidopsis promoted anthocyanin biosynthesis under 1% sucrose, but inhibited this process under 9% sucrose (Supplementary Figs S4C, D, S5A). Similarly, compared with 1% sucrose, 12% sucrose inhibited anthocyanin biosynthesis in *MdSnRK1.1* transgenic apple calli (Fig. 2A, B; Supplementary S4A, B). Therefore, there is a threshold sucrose concentration for the induction of anthocyanin biosynthesis in apple calli and Arabidopsis. Sucrose at concentrations higher and lower than this threshold inhibits the accumulation of anthocyanins.

The demand for and utilization of sucrose vary with plant species, developmental stages, and environment cues, which may affect the SnRK1 activity (Rolland *et al.*, 2006; Baena-González and Sheen, 2008). Compared with zero sucrose, 9% sucrose inhibits anthocyanin biosynthesis in *MdSnRK1.1* transgenic Arabidopsis but promotes this process in transgenic apple calli (Supplementary Fig. S4), suggesting that apple calli have a higher sugar tolerance than Arabidopsis. This also explains the discrepancy between our data concerning *MdSnRK1.1* ectopic transgenic Arabdidopsis and those of a previous study about *AtSnRK1.1* overexpression in transgenic Arabidopsis (Baena-González *et al.*, 2007; Supplementary Fig. S5).

In Arabidopsis, SnRK1 is involved in various life processes by phosphorylating its specific substrates (Zhang *et al.*, 2008). The substrates not only include the key metabolic enzymes such as sucrose phosphate synthase, nitrate reductase, and HMG-CoA reductase, but also the TFs such as FUS3 (Sugden *et al.*, 1999; Harthill *et al.*, 2006; Tsai and Gazzarrini, 2012). The tomato SlSnRK1 phosphorylates the βC1 protein that is the pathogenicity determinant and a suppressor of RNA silencing (Shen *et al.*, 2011). SnRK1 is also involved in the innate antiviral defense by interacting with AL2 and L2, which are the geminivirus pathogenicity proteins (Hao *et al.*, 2003). SnRK1 interacts with and phosphorylates adenosine kinase (ADK) to maintain energy homeostasis and to regulate the responses to biotic and abiotic stresses (Mohannath *et al.*, 2014). In this study, it was found that MdSnRK1.1 interacted with and phosphorylated the MdJAZ18 protein in apple (Figs 3A–C, 4A), which further promoted its degradation (Fig. 4B–H). In mammals, AMPK is structurally and functionally analogous to SnRK1 in higher plants. It phosphorylates TXNIP (thioredoxin-interacting protein) and accelerates its degradation upon energy stress (Wu *et al.*, 2013). In our study, MG132 treatment eliminated the effect of MdSnRK1.1-induced MdJAZ18 phosphorylation on the degradation of MdJAZ18 protein, suggesting that the degradation depends on the 26S proteasome (Fig. 4B, C).

The JAZ proteins act as repressors in the JA signaling pathway. They are recruited by the F-box protein (COI1) for ubiquitination and subsequent degradation through the 26S proteasome in response to JA signal, thereby releasing the downstream JA-responsive factors (Qi *et al.*, 2011; Wasternack and Hause, 2013). The JAZ proteins contain two domains, which are the N-terminal ZIM domain and the C-terminal Jas domain (Wasternack and Hause, 2013). The conserved ZIM (TIFY) domain of JAZ proteins is responsible for JAZ dimerization and interaction with the co-repressors NINJA (Pauwels *et al.*, 2010). In this study, it was found that the N-terminal ZIM domain of MdJAZ18 protein is essential for its interaction with MdSnRK1.1 (Fig. 3A). Hence, it is possible that MdSnRK1.1 competes with NINJA to interact with MdJAZ18, which consequently results in JA responses and therefore links sugar and JA signaling pathways.

**Fig. 6.**
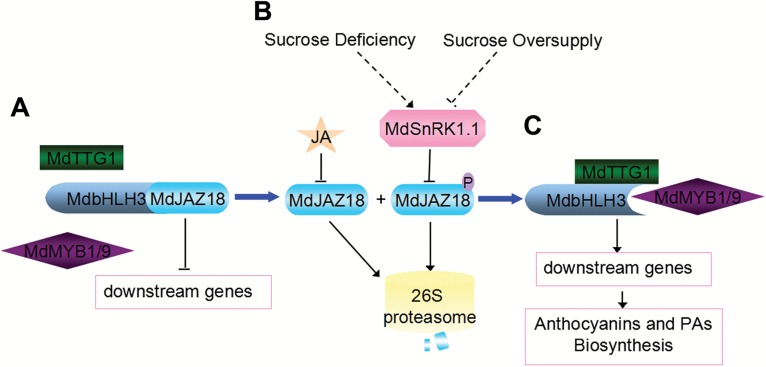
Proposed model for the involvement of MdSnRK1.1 in sucrose- and JA-regulated anthocyanin and PA biosynthesis in apple. (A) The interaction of MdJAZ18 with the bHLH transcription factor MdbHLH3 represses the transcriptional function of the MBW complex to activate downstream genes. (B) Under sucrose deficiency, MdSnRK1.1 phosphorylates MdJAZ18, and then accelerates its degradation through the 26S proteasome. However, under sucrose oversupply, this process is inhibited. Sucrose and JA have a synergistic effect on the degradation of MdJAZ18 protein. (C) Once the degradation of MdJAZ18 protein is completed, MdbHLH3 is released and then interacts with MdMYBs (MdMYB1 and MdMYB9) and MdTTG1 to form an active MBW complex, or bind to the promoters of MdMYB (*MdMYB1* and *MdMYB9*) genes to regulate the biosynthesis of anthocyanins and PAs.

In Arabidopsis, JAZ proteins directly interact with bHLH TFs (GL3, EGL3, and TT8) and MYB TFs (MYB75 and GL1). These bHLH and MYB TFs are essential components of the MBW complex to mediate anthocyanin accumulation (Qi *et al.*, 2011). In apple, MdJAZ18, MdJAZ1, and MdJAZ19 interact with the MdbHLH3 protein. MdbHLH3 not only improves the transcription level of *MdMYB1*, *MdMYB9*, and *MdMYB11*, but also interacts with three MdMYB proteins to activate the expression of *MdANS*, *MdANR*, and *MdLAR* to regulate anthocyanin and PA biosynthesis (Xie *et al.*, 2012; An *et al.*, 2015). Therefore, JAs stimulate anthocyanin and PA accumulation by up-regulating the expression of these flavonoid biosynthetic genes (Qi *et al.*, 2011; Wasternack and Hause, 2013; An *et al.*, 2015). Therefore, our findings support a model in which MdSnRK1.1 promotes the accumulation of anthocyanins and PAs by phosphorylating and degrading MdJAZ18 to release MdbHLH3 in response to sucrose deficiency, which subsequently activates the expression of the regulatory and structural genes (Fig. 6). This mechanism explains the long known fact that JA acts together with sucrose to improve anthocyanin biosynthesis (Loreti *et al.*, 2008). The integration of sugar and JA signals was verified by the interaction between MdSnRK1.1 and MdJAZ18 proteins, which controls anthocyanin and PA accumulation through the modulation of the function of the MBW complex in plants.

Besides anthocyanin biosynthesis, JAs modulate male and female fertility, root growth, and trichome formation (Stintzi, 2000; Pauwels *et al.*, 2010; Qi *et al.*, 2011). Furthermore, they also regulate a wide range of defense processes, such as pathogen infection, insect attack, UV damage, wounding, and many other abiotic stresses (Nibbe *et al.*, 2002; Xiao *et al.*, 2004; Schilmiller and Howe, 2005; Wasternack and Hause, 2013). JAs activate the defense responses against herbivorous insects and necrotrophic pathogens through regulating the expression of *VSP2* and *PDF1.2* (Wasternack and Hause, 2013). Similarly, sugars regulate stress-inducible and pathogenesis-related genes (Sadka *et al.*, 1994; Loreti *et al.*, 2008). In plants, elevated levels of cellular sugar increase the expression level of genes associated with defense responses (Price *et al.*, 2004). The sensitivity to virus attack is increased in the transgenic tobacco plants which contain an antisense sequence of the Arabidopsis *SnRK1* gene (Hao *et al.*, 2003; Polge and Thomas, 2007). For the first time, our findings and the model concerning the interaction between MdSnRK1.1 and MdJAZ18 shed light on the molecular mechanism by which the crosstalk of sugar and JA signaling regulates defense against various abiotic and biotic stresses.

## Supplementary data

Supplementary data are available at *JXB* online.

Fig. S1. Protein sequence comparison of MdSnRK1s or MdSnRK1.1 with AtSnRK1.1.

Fig. S2. Phylogenic tree of Arabidopsis and apple SnRK1 protein.

Fig. S3. Generation of *MdSnRK1.1* and asMdSnRK1.1 transgenic plants.

Fig. S4. MdSnRK1.1 mediates anthocyanin accumulation in response to sucrose.

Fig. S5. Ectopic expression of the *MdSnRK1.1* gene enhances sucrose sensitivity and promotes anthocyanin accumulation in Arabidopsis under 1% sucrose.

Fig. S6. Phylogenic tree of Arabidopsis and apple JAZ proteins.

Fig. S7. Y2H assay to test interactions of SnRK1.1 with the MdJAZs proteins in apple and AtJAZ3 in Arabidopsis.

Fig. S8. MdSnRK1.1 fails to phosphorylate MdJAZ12 *in vitro*.

Fig. S9. Sucrose influences MdSnRK1.1-mediated MdJAZ18 degradation.

Fig. S10 GUS activity of MdJAZ18–GUS transgenic calli in response to JA.

Fig. S11. Generation of transgenic calli that transform MdJAZ18 in the MdSnRK1.1 and asMdSnRK1.1 background, and which express MdSnRK1.1 and asMdSnRK1.1 in MdJAZ18–GUS transgenic calli.

Fig. S12. The expression levels of *MdUF3GT* and *MdANS* genes in the WT and transgenic calli in Fig. 5A.

Table S1. Primers used for qRT-PCR and vector construction.

## Supplementary Material

supplementary_figures_S1_S12_table_S1
