# Supplementary material for: MdSnRK1.1 interacts with MdJAZ18 to regulate sucrose-induced anthocyanin and proanthocyanidin accumulation in apple
Source: J Exp Bot. 2017 May 25;68(11):2977–90. doi: 10.1093/jxb/erx150 (PMC5853841; doi:10.1093/jxb/erx150)
Supplement: supplementary_figures_S1_S12_table_S1 [file erx150_suppl_supplementary_figures_s1_s12_table_s1.pdf]

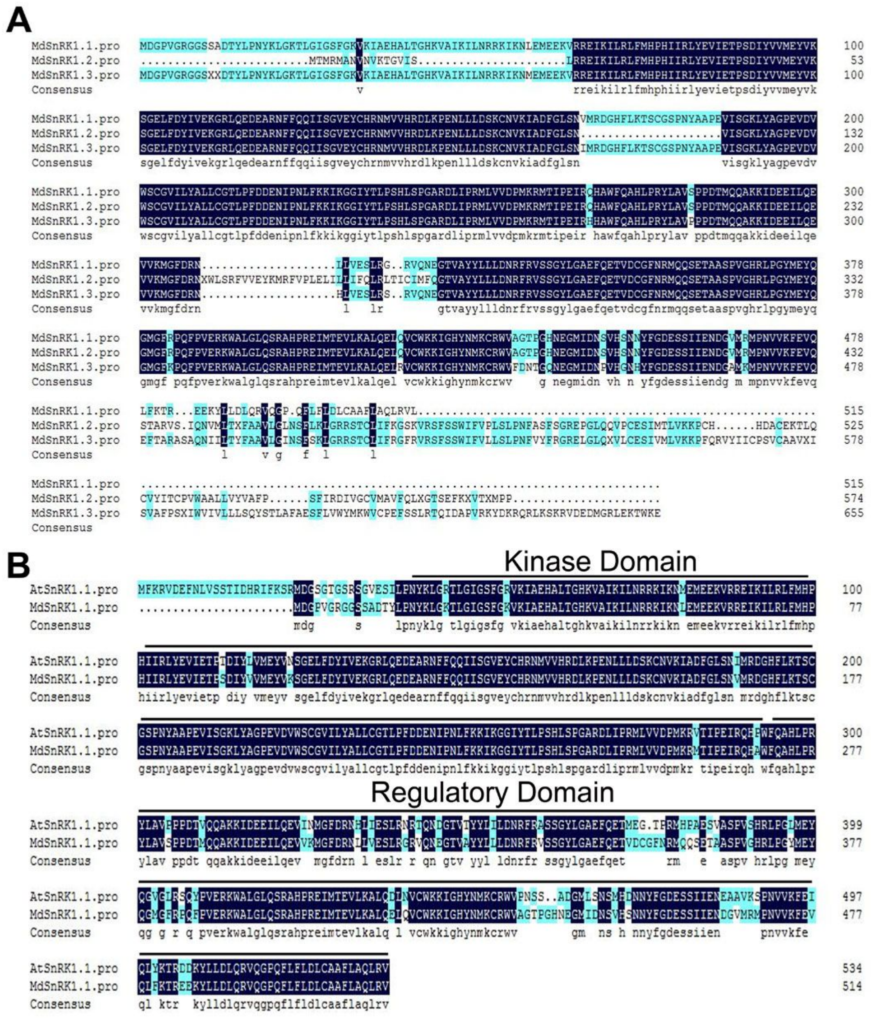

Figure S1. Proteins sequence comparison about MdSnRK1s, or MdSnRK1.1 with AtSnRK1.1. In (A-B) the navy blue bars shows identical amino acids, the residues are different. The sequences were divided into two functional domain, kinase domain and regulation domain in (B).

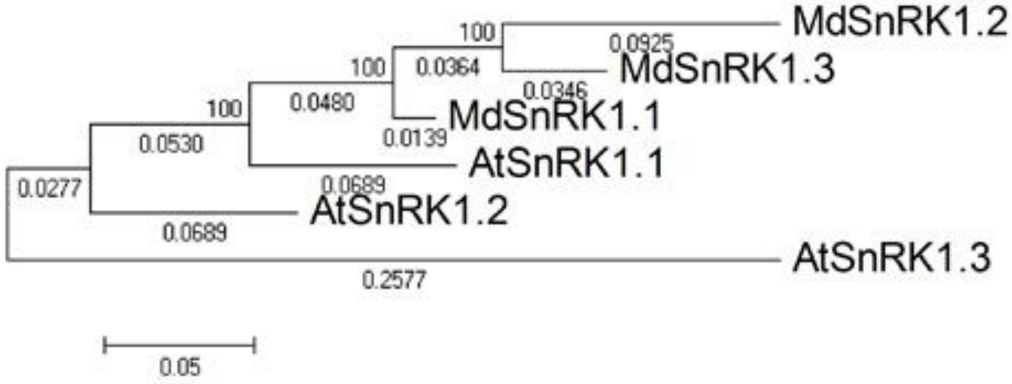

Figure. S2 Phylogeny tree of Arabidopsis and apple SnRK1s proteins. The alignment was conducted with the Mega4 using the neighbor-joining method.

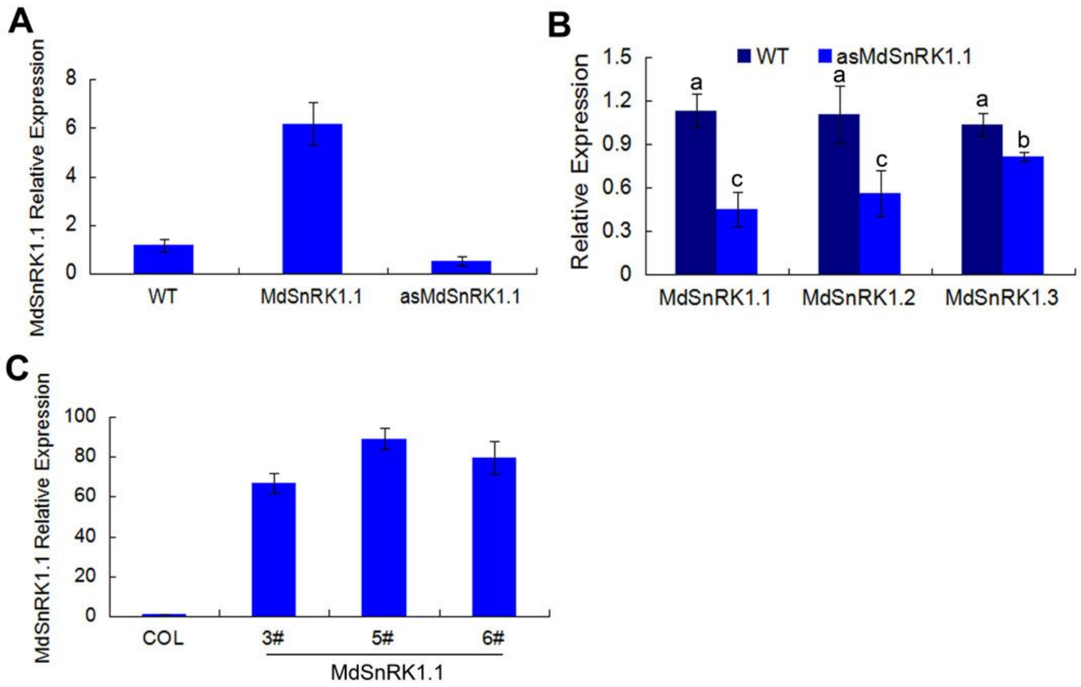

Figure S3. Generation of *MdSnRK1.1* and asMdSnRK1.1 transgenic plants. (A) qRT-PCR analysis of *MdSnRK1.1* expression in WT and the transgenic calli. (B) qRT-PCR analysis of MdSnRK1s expression in WT and asMdSnRK1.1 transgenic calli. (C) Transcript level of *MdSnRK1.1* by qRT-PCR in the wild-type Arabidopsis ('Columbia' ecotype) and three transgenic Arabidopsis (MdSnRK1.1-3, MdSnRK1.1-5 and MdSnRK1.1-6). *18s* was used as the internal control. Error bars represent SD based on three biological replicates. In B, statistical significance was calculated with DPS software of LSD test, $p<0.05$.

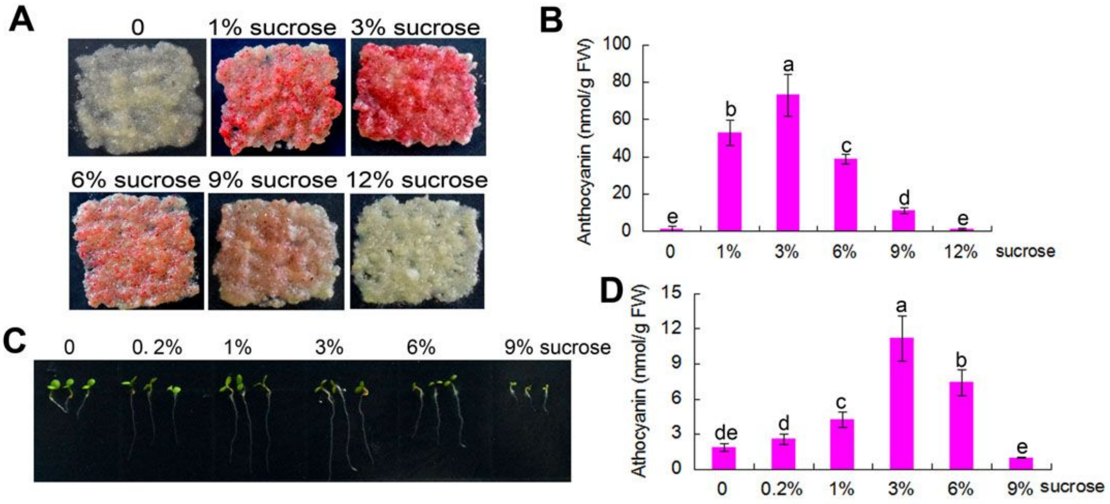

Figure S4. MdSnRK1.1 mediates anthocyanin accumulation in response to sucrose. (A-D) Coloration (A and C) and anthocyanin contents (B and D) of MdSnRK1.1 transgenic apple calli (A-B) and transgenic Arabidopsis (C-D) treated with different sucrose as indicated. In (B) and (D), error bars represent SD. Statistical significance was calculated with DPS software of LSD test, $P<0.05$.

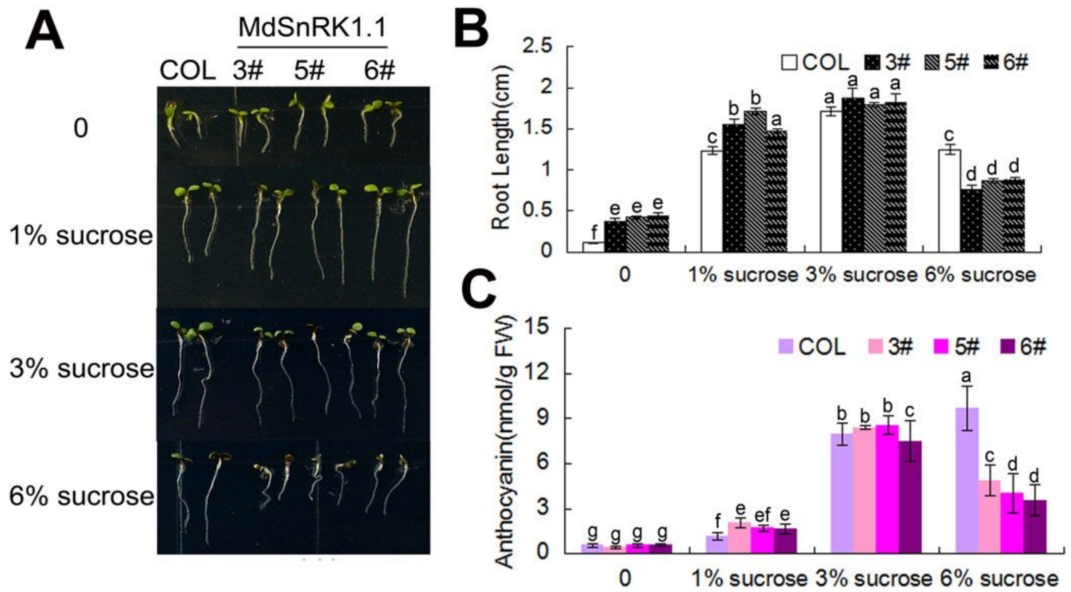

Figure S5. Ectopic expression of *MdSnRK1.1* gene enhances sucrose sensitivity and promotes anthocyanin accumulation in *Arabidopsis* under 1% sucrose condition. (A)Phenotype of wild-type Columbia (Col) and three transgenic lines (MdSnRK1.1-3, MdSnRK1.1-5 and MdSnRK1.1-6) seedlings grown with various sucrose (0, 1%, 3% and 6%). (B) Root lengths of *Arabidopsis* seedlings in (A). (C) Anthocyanin contents in (A) seedlings. In (C), FW, fresh weight. In (B) and (C), error bars represent SD. Statistical significance was calculated with DPS software of LSD test, $p<0.05$.

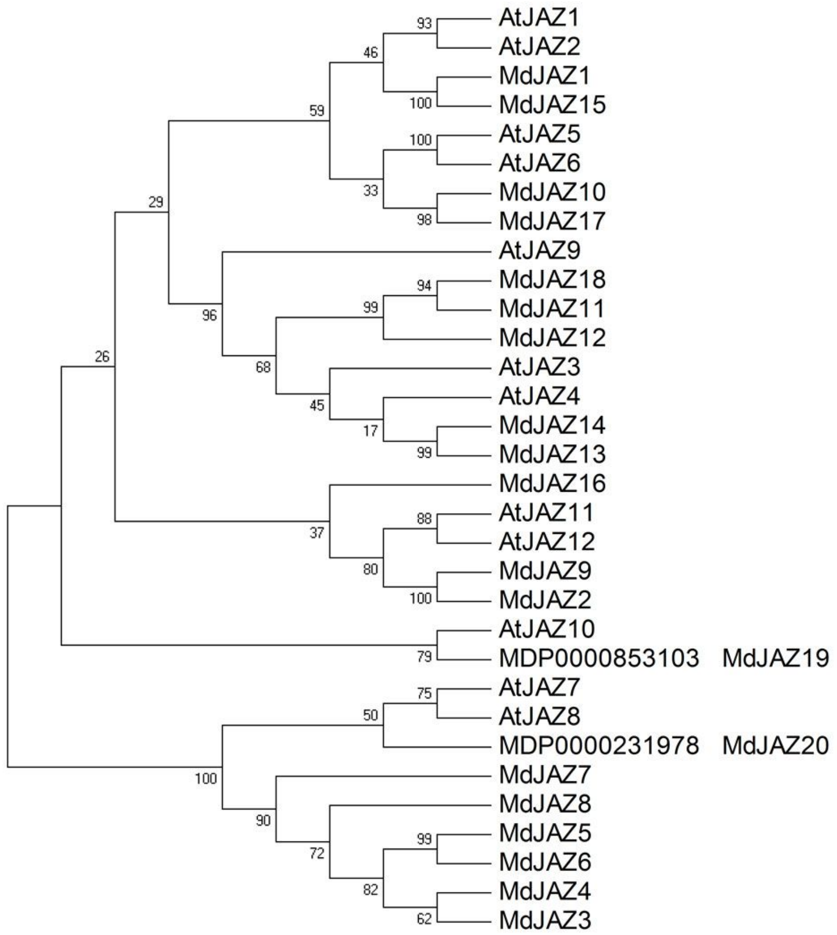

Figure. S6 Phylogeny tree of *Arabidopsis* and apple JAZs proteins. The alignment was conducted with the Mega4 using the neighbor-joining method.

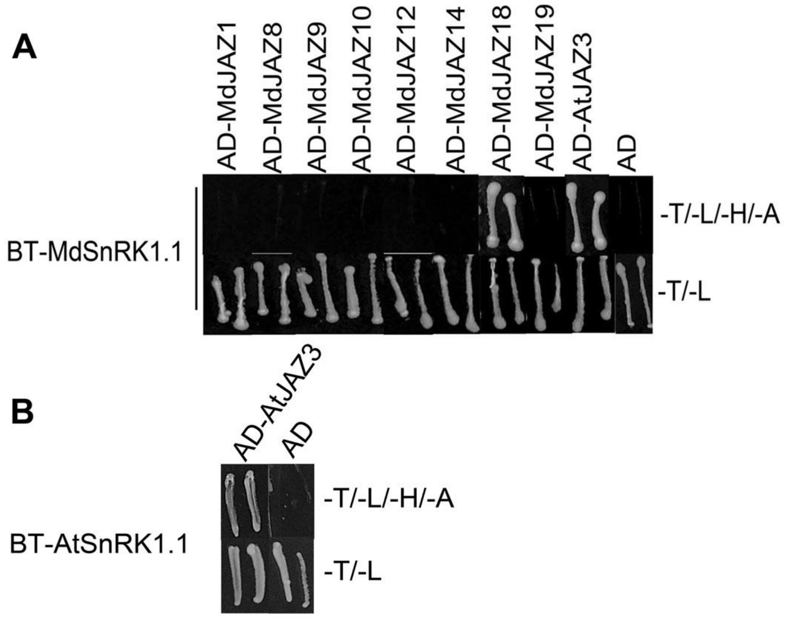

Figure S7. Y2H assay to test interactions of SnRK1.1 with the MdJAZs proteins in apple and AtJAZ3 in *Arabidopsis*. (A) AD-MdJAZs and AD-AtJAZ3 were to verified the interaction with BT-MdSnRK1.1 in yeast cells. AD was as negative control. (B) AD-AtJAZ3 was to verified the interaction with BT-AtSnRK1.1 in yeast cells. AD was as negative control.

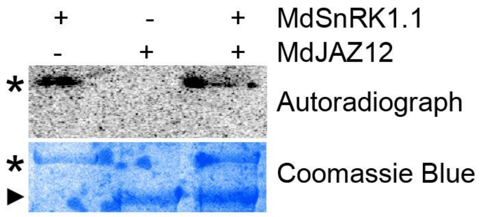

Figure S8. MdSnRK1.1 fails to phosphorylate MdJAZ12 *in vitro*. The asterisk indicated the autophosphorylation of the purified HIS-MdSnRK1.1 in autoradiogram. The asterisk and triangle referred to protein loading of HIS-MdSnRK1.1 and HIS-MdJAZ12, respectively, in coomassie blue staining.

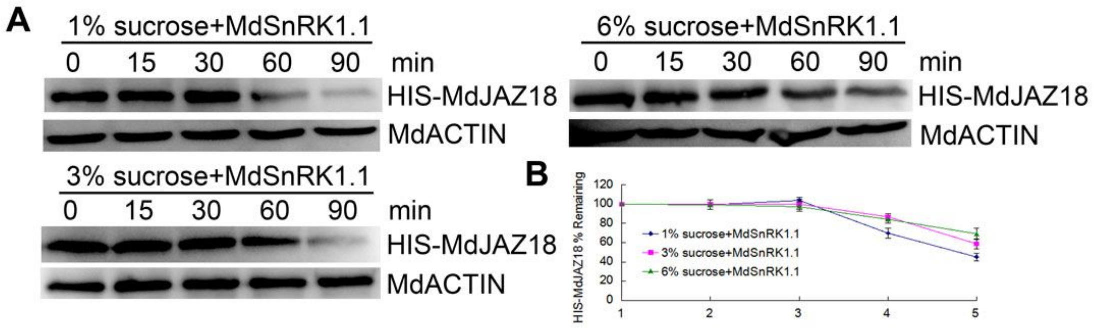

Figure S9. Sucrose influences MdSnRK1.1-mediated MdJAZ18 degradation. (A-B) The purified HIS-MdJAZ18 were incubated with the total proteins extracted from MdSnRK1.1 transgenic apple calli treated with different sucrose (1%, 3% or 6%). The samples were harvested at the denoted time. MdACTIN was used as internal reference. In (B), error bars represent SD.

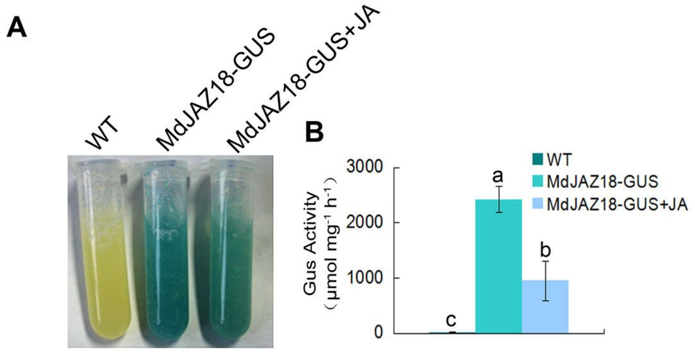

Figure. S10 Gus activity of MdJAZ18-GUS transgenic calli in response to JA.

The GUS staining of WT and MdJAZ18-GUS calli treated with or without JA in (A) image, correspondent GUS activity in (B) image. Error bars represent SD based on three biological replicates. Statistical significance was calculated with DPS software of LSD test, $P<0.05$.

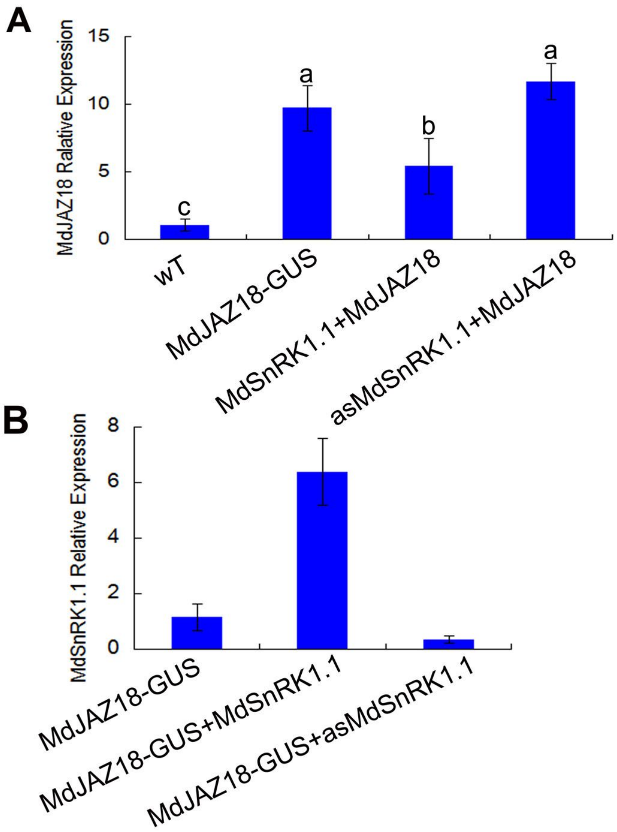

Figure S11. Generation of transgenic calli that transforming MdJAZ18 in MdSnRK1.1 and asMdSnRK1.1 background, and expressing MdSnRK1.1 and asMdSnRK1.1 in MdJAZ18-GUS transgenic calli. (A) The expression levels of *MdJAZ18* in WT, MdJAZ18-GUS, MdSnRK1.1+MdJAZ18 and asMdSnRK1.1+MdJAZ18 transgenic calli were analyzed by qRT- PCR. (B) Transcript levels of *MdSnRK1.1* in WT, MdJAZ18-GUS+MdSnRK1.1 and MdJAZ18-GUS+asMdSnRK1.1 transgenic calli were revealed by qRT-PCR. *18s* was used as the internal control. Error bars represent SD based on three biological replicates. Statistical significance was calculated with DPS software of LSD test, P<0.05.

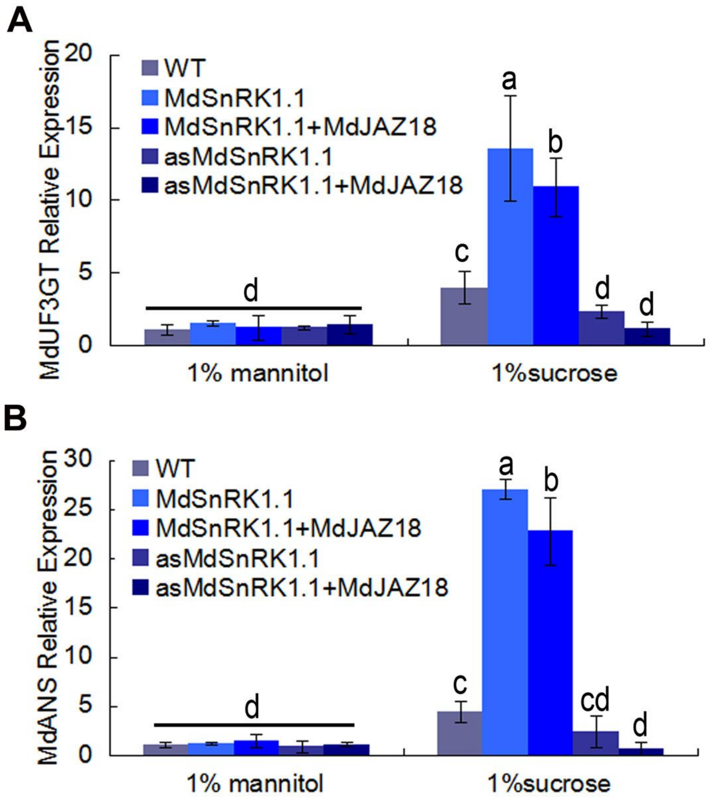

Figure S12. The expression levels of *MdUF3GT* (A) and *MdANS* (B) genes in WT and transgenic calli in figure 5(A). *18s* was used as the internal control. Error bars represent SD based on three biological replicates. Statistical significance was calculated with DPS software of LSD test, P<0.05.

Table S1. List of primers used for RT-PCR and vector construction.

Name	Primers sequences
MdPAL (MDP0000261492)	(RT)-F: GTGAGGGAGGAGTTGGGAGGAG<br>(RT)-R:CTCCTCCCAACTCCTCCCTCAC
MdCHI (MDP0000759336)	(RT)-F: GCTACAAATGCGGTGATAG<br>(RT)-R:CGCCTCCACTACAACCTCC
MdCHS (MDP0000686666)	(RT)-F: GGCAAGTGCTGTCGGATT<br>(RT)-R: CCCAAAGAAATAACCACAAG
MdF3H (MDP0000323864)	(RT)-F: GCCGATCACCTACACCGAG<br>(RT)-R:GTACAAGAAGTGGGAAGGC
MdDFR (MDP0000494976)	(RT)-F:GTTGAGGGAGATAGGGTTTGAG<br>(RT)-R:GGTAAATGTAAAACAATAGAGAGG
MdANR (MDP0000271553)	(RT)-F:TCAACAAAAGATACCCCCAG<br>(RT)-R:GATAGCTAGCTCGATACATGC
MdUF3GT (MDP0000405936)	(RT)-F: GGAAGTGGTTTTGTCGCCTG<br>(RT)-R:CATTATTATTGAGCAACGAACAGC
MdANS (MDP000024061)	(RT)-F:GGAGAAGATCATCCTTAAGCCA<br>(RT)-R:CTAAGATATATCATACCAACTATGCC
MdFLS (MDO0000183682)	(RT)-F:GGATAAGACAAGAATCTCATGGC<br>(RT)-R:CACACCACTCACAACTTTACC
MdbHLH33 (MDP0000309179)	(RT)-F: GCCTGCACTTCACTGAAATC<br>(RT)-R: GTTGTGTAGTTGACAATGACC
MdMYB9 (MDP0000210851)	(RT)-F: GATGAGGCAATGATAAATGACG<br>(RT)-R: TTAGACTACAACATTTTCTTGGG
MdMYB1 (MDP0000807341)	(RT)-F:GAAAGAGCTGCATATCCCAG<br>(RT)-R: CTATTCTTCTTTTGAATGATTCC
MdMYB11(MDP0000437717)	(RT)-F: GTCGATTTCTCTGTGCTCTATAAC<br>(RT)-R: TTAATTATCTACGAGCCAGCAGTC
MdbHLH3 (MDP0000225680)	(RT)-F: CCAAAAATGGCTGCACCGCC<br>(RT)-R: CTTGATAGCTCCATTATAGTACCC
MdSnRK1.1 (MDP0000191788)	(RT)-F: AGAGACCGATTTCGCCTTGC<br>(RT)-R: CTTTATGTCCAGTTAATGC
MdJAZ18 (MDP0000757701)	(RT)-F: CGTGGAACAATGTCAAGAC<br>(RT)-R: CAGCACTAGAGGAGTTAGAAC
MdSnRK1.1 (PRI) (MDP0000191788)	F: GTCGACATGGATGGACCTGTTG<br>R: GGATCCCAAGGACGCGAAGTTG
MdSnRK1.1 (PXSN) (MDP0000191788)	F: AATGGATGGACCTGTTG<br>R:CTACAAGGACGCGAAGTTG
MdSnRK1.1 (IL) (MDP0000191788)	F: GAATTCATGGATGGACCTGTTG<br>R: GTCGACCTACAAGGACGCGAAGTTG
MdSnRK1.1 (BT) (MDP0000191788)	F:GAATTCATGGATGGACCTGTTG<br>R: GGATCCAAGGACGCGAAGTTG
MdSnRK1.1 (PET32a) (MDP0000191788)	F: GGATCCATGGATGGACCTGTTG<br>R: GTCGACAAGGACGCGAAGTTG

MdSnRK1.1$^{1-271}$ (BT) (MDP0000191788)	F: GAATTCATGGATGGACCTGTTG<br>R: GGATCCGAACCATGCATGCTGAC
MdSnRK1.1$^{272-516}$ (BT) (MDP0000191788)	F: GAATTCCGAGGAAGATGAGCCTG<br>R:GGATCCAAGGACGCGAAGTTG
asMdSnRK1.1 (PRI) (MDP0000191788)	F: GTCGACATGGATGGACCGGTTGG<br>R:GGATTCGCATCAAATTTAAGGGGAAC
asMdSnRK1.1 (PXSN) (MDP0000191788)	F: AATGGATGGACCGGTTGG<br>R: GCATCAAATTTAAGGGGAAC
MdJAZ18 (PRI) (MDP0000757701)	F: GTCGACATGGAGAGAGATTTCTTGGG<br>R: GAATTCCACCTTTCCTTGCGCTTCTCT
MdJAZ18 (PXSN) (MDP0000757701)	F: AATGGAGAGAGATTTCTTGGG<br>R: TCACCTTTCCTTGCGCTTCTCT
MdJAZ18 (AD/PGEX) (MDP0000757701)	F: GAATTCATGGAGAGAGATTTCTTGGG<br>R: GTCGACCACCTTTCCTTGCGCTTCTCT
MdJAZ18$^{1-262}$ (AD) (MDP0000757701)	F: GAATTCATGGAGAGAGATTTCTTGGG<br>R: GTCGACAGAAAGAGCAGGTGGGGGTA
MdJAZ18$^{256-343}$ (AD) (MDP0000757701)	F: GAATTCCCCCCACCTGCTCTTTCTA<br>R: GTCGACCCTTTCCTTGCGCTTCTCTA
18S	F: ACACGGGAGGTAGTGACAA<br>R: CCTCCAATGGATCCTCGTTA
